# Clinical spectrum and IgG subclass analysis of anti-myelin oligodendrocyte glycoprotein antibody-associated syndromes: a multicenter study

**DOI:** 10.1007/s00415-017-8635-4

**Published:** 2017-10-23

**Authors:** Sara Mariotto, Sergio Ferrari, Salvatore Monaco, Maria Donata Benedetti, Kathrin Schanda, Daniela Alberti, Alessia Farinazzo, Ruggero Capra, Chiara Mancinelli, Nicola De Rossi, Roberto Bombardi, Luigi Zuliani, Marco Zoccarato, Raffaella Tanel, Adriana Bonora, Marco Turatti, Massimiliano Calabrese, Alberto Polo, Antonino Pavone, Luisa Grazian, GianPietro Sechi, Elia Sechi, Daniele Urso, Rachele Delogu, Francesco Janes, Luciano Deotto, Morena Cadaldini, Maria Rachele Bianchi, Gaetano Cantalupo, Markus Reindl, Alberto Gajofatto

**Affiliations:** 1 Department of Neuroscience, Biomedicine and Movement Sciences, Neurology Unit, University of Verona, Verona, Italy; 2 Clinical Department of Neurology, Medical University of Innsbruck, Innsbruck, Austria; 3 Multiple Sclerosis Centre, Spedali Civili of Brescia, Montichiari, Brescia Italy; 4 Neurology Unit, St Bassano Hospital, Bassano del Grappa, Vicenza Italy; 5 Neurology Unit, ULSS 2 Marca Trevigiana, Ca’ Foncello Hospital, Treviso, Italy; 6 Neurology Unit, O.S.A, Padua, Italy; 7 Neurology Unit, S. Chiara Hospital, Trento, Italy; 8 Ophthalmology Unit, AOUI Verona, Verona, Italy; 9 Neurology Unit, Mater Salutis Hospital, Legnago, Verona Italy; 10 Neurology Unit, Garibaldi Hospital, Catania, Italy; 11 Pediatric Unit, ULSS 2 Marca Trevigiana, Ca’ Foncello Hospital, Treviso, Italy; 12 Neurology Unit, Department of Clinical and Experimental Medicine, University of Sassari, Sassari, Italy; 13 Neurology Unit, Department of Neuroscience, ASUIUD, Udine, Italy; 14 Neurology A Unit, AOUI Verona, Verona, Italy; 15 Multiple Sclerosis Centre of Este, Padua, Italy; 16 Neurology Unit, AAS2 Bassa Friulana-Isontina, Gorizia, Italy; 17 Child Neurology, University of Verona, Verona, Italy

**Keywords:** Anti-myelin oligodendrocyte glycoprotein (MOG) antibodies, Neuromyelitis optica spectrum disorders (NMOSD), Multiple sclerosis (MS), Optic neuritis, Myelitis, Acute disseminated encephalomyelitis (ADEM)

## Abstract

Anti-myelin oligodendrocyte glycoprotein antibodies (MOG-Ab) recently emerged as a potential biomarker in patients with inflammatory demyelinating diseases of the central nervous system. We here compare the clinical and laboratory findings observed in a cohort of MOG-Ab seropositive and seronegative cases and describe IgG subclass analysis results. Consecutive serum samples referred to Verona University Neuropathology Laboratory for aquaporin-4 (AQP4)-Ab and/or MOG-Ab testing were analysed between March 2014 and May 2017. The presence of AQP4-Ab was determined using a cell-based assay. A live cell immunofluorescence assay was used for the detection of MOG-IgG and IgG subclass analysis. Among 454 analysed samples, 29 were excluded due to AQP4-Ab positivity or to the final demonstration of a disorder not compatible with MOG-Ab. We obtained clinical data in 154 out of 425 cases. Of these, 22 subjects resulted MOG-Ab positive. MOG-Ab positive patients were mainly characterised by the involvement of the optic nerve and/or spinal cord. Half of the cases presented relapses and the recovery was usually partial. Brain MRI was heterogeneous while short lesions were the prevalent observation on spinal cord MRI. MOG-Ab titre usually decreased in non-relapsing cases. In all MOG-IgG positive cases, we observed IgG1 antibodies, which were predominant in most subjects. IgG2 (5/22), IgG3 (9/22) and IgG4 (3/22) antibodies were also detectable. We confirm that MOG-Ab-related syndromes have distinct features in the spectrum of demyelinating conditions, and we describe the possible role of the different IgG subclasses in this condition.

**Electronic supplementary material:**

The online version of this article (doi:10.1007/s00415-017-8635-4) contains supplementary material, which is available to authorized users.

## Introduction

Inflammatory demyelinating diseases (IDD) represent a spectrum of heterogeneous disorders affecting the central nervous system (CNS). Multiple sclerosis (MS) with its variants and neuromyelitis optica spectrum disorders (NMOSD) that preferentially involve the spinal cord and the optic nerve are the most defined forms. The identification of autoantibodies directed against aquaporin-4 (AQP4-Ab) in the serum of the majority of NMOSD patients has significantly facilitated the distinction from MS and other conditions [1, 2]. However, in about 10–40% of NMOSD cases, AQP4-Ab are not detected causing diagnostic uncertainty [3–6], although criteria for seronegative NMOSD have been recently proposed [7]. Several recent studies have identified the presence of anti-myelin oligodendrocyte glycoprotein antibodies (MOG-Ab) in the serum of children and adults with various IDD, including AQP4-Ab negative NMOSD, acute disseminated encephalomyelitis (ADEM), idiopathic optic neuritis, idiopathic myelitis, and atypical MS [8–32]. However, the clinical characteristics of MOG-Ab positive patients have yet to be entirely clarified in terms of diagnostic classification, prognosis and treatment. Furthermore, the different techniques currently used for the detection of MOG-Ab can create diagnostic discrepancies. In particular, the demonstration of humoral immune reaction against conformational MOG epitopes seems in favour of the use of live cell-based assay (CBA). To avoid false positive samples without losing low-titre true positives, some groups established MOG-IgG high-titre cut-off, while others prefer to measure IgG1 MOG-Ab according to previous findings that MOG-Ab belong mainly to the IgG1 subclass [19, 29, 33].

In this study, we analysed MOG-Ab serostatus in a large series of patients with IDD with the aim of assessing the diagnostic utility of MOG-Ab testing in the clinical practice. We report on clinical and paraclinical characteristics of 22 patients with serological evidence of MOG-Ab and compare them with a group of MOG-Ab negative cases. Moreover, we studied IgG MOG-Ab subclasses in all subjects to understand if autoantibody response can differ between patients and to compare different diagnostic techniques.

## Methods

All the data analysed in the present study were collected as part of the standard clinical practice at study centres. Since no additional research procedures were performed, approval of the local ethics committee was not needed. All patients consented to diagnostic procedures and biological sample storage at Verona Neuropathology Laboratory.

### Study subjects

**Fig. 1:**
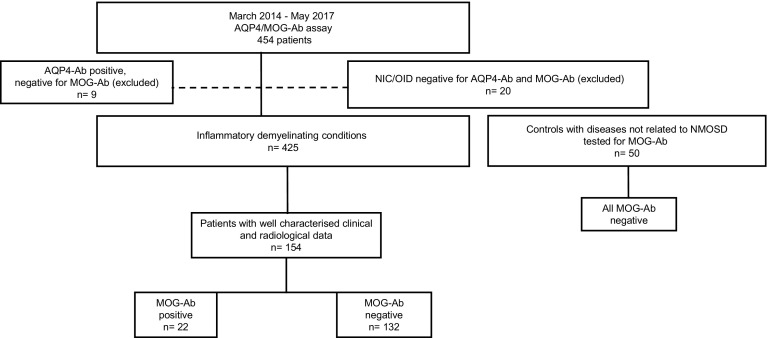
Patient cohort. Of the 454 serum samples that were referred to the laboratory of Neuropathology, University Hospital of Verona for the analysis of AQP4-Ab/MOG-Ab between March 2014 and May 2017, nine resulted AQP4-Ab positive and MOG-Ab negative and were excluded from further analysis. We also excluded 20 subjects that received a final diagnosis of non-inflammatory neurological conditions or other defined inflammatory disorders. In 154 out of 425 included subjects, the clinical and paraclinical data had been well-characterised. Among the final cohort, 132 patients resulted as MOG-Ab negative and 22 MOG-Ab positive, at different titres (1:160 in 5 cases, 1:320 in 4, 1:640 in 7, 1:1280 in 2, 1:2560 in 2, 1:10,240 in 1 and 1:81,920 in 1 case). An additional group of 50 control samples were also tested for MOG-Ab, and resulted negative

We identified patients referred by the treating physicians for serum AQP4/MOG-Ab assay to the Laboratory of Neuropathology, University Hospital of Verona, Italy, between March 2014 and May 2017. Of the 454 consecutive serum samples that were analysed, nine resulted AQP4-Ab positive and MOG-Ab negative, and were excluded from further analysis. We also excluded 20 subjects that received a final diagnosis of non-inflammatory neurological disorders (NIC) or other defined inflammatory disorders (OID). Among 425 included subjects, 403 resulted as MOG-Ab negative and 22 MOG-Ab positive. Clinical, cerebrospinal fluid (CSF) and MRI data obtained at onset and during the follow-up were adequately available for 132 seronegative cases and for all the MOG-Ab positive subjects. An additional group of 50 anonymised serum samples from control patients with neurological conditions not related to NMOSD, including peripheral neuropathies, suspected lysosomal storage disorders and stroke were also tested for MOG-Ab (Fig. 1). The cohort was composed mainly of adults; only four MOG-Ab negative subjects and two MOG-Ab positive cases were considered paediatric at onset.

### Clinical data and diagnosis

For all 154 included subjects, demographic and clinical data were collected from medical records and report forms referring physicians need to fill out upon AQP4/MOG-Ab testing request at Verona Neuropathology Laboratory. The clinical course was classified as monophasic when only one clinical acute/subacute event occurred, relapsing in patients with one or more relapses or gradually evolving in those with an insidiously worsening course over time. A “relapse” was defined according to McDonald criteria [34]. At last follow-up visit, at least 1 month after the index event, recovery was considered complete if neurological examination was normal and no symptoms were reported (expanded disability status scale—EDSS—score 0 or equal to baseline value), absent if no improvement was observed (EDSS score at last follow-up ≥ EDSS score at nadir), partial in all the other cases.

According to data available at the time of MOG-Abs testing, clinical diagnosis was defined according to nine diagnostic categories: (1) clinically isolated syndrome (CIS) [35]; (2) MS [34]; (3) NMOSD [7]; (4) chronic relapsing inflammatory optic neuropathy (CRION) [36, 37]; (5) ADEM [38]; (6) idiopathic optic neuritis (ON); (7) idiopathic myelitis (MY); (8) both MY and ON; (9) other demyelinating disorders. Idiopathic ON and/or MY were defined as one or more episodes of acute/subacute optic neuropathy and/or myelopathy of inflammatory origin (based on clinical, radiological and/or CSF evidence) not fulfilling diagnostic criteria for MS, NMOSD, and ADEM and not attributable to other causes. Other demyelinating disorders were characterised by inflammatory conditions defined by clinical, CSF and radiological evidence, with multifocal lesions not included in the disorders previously mentioned. Based on the revision of all available data at the time of last follow-up visit, two investigators (AG, SM) blinded to MOG-Ab assay result classified each patient according to the diagnostic criteria mentioned above.

### Laboratory data

Serological and CSF analysis were performed in the local general laboratory of participating centres. The presence and pattern of oligoclonal bands (OB) were analysed according to international guidelines [39].

### MRI data

Brain and spinal cord MRI data were collected from scans obtained within 3 months from the blood drawn for AQP4/MOG-Ab analysis. All MRIs analyses of patients followed at Verona University Hospital were reviewed by a single reader (AG), while MRI performed at other centres were reviewed by the treating neurologist of each case. MRIs were obtained with different ≥ 1.5 Tesla scanners and included at least axial and sagittal (or volumetric) T2 and fluid-attenuated inversion recovery (FLAIR) sequences with slice thickness ≤ 3 mm.

The presence of any abnormal finding, the number of focal lesions, gadolinium-enhancing lesions, and the involvement of the optic nerve were reported. Spinal cord lesions were categorized according to the involved anatomical level and longitudinal extension ≥ 3 vertebral segments or less.

### AQP4-Ab and MOG-Ab assays

Blood samples were collected using plastic tubes without anti-coagulant (vacuum tube GEL & CLOT ACT, 5 mL REF 10020, Vacutest Kima, Arzergrande, Padova, Italy), centrifuged, and stored at −80 °C. In all cases, the presence of serum AQP4-Ab was analysed using a commercially available cell-based assay (Anti-Aquaporin-4 IIFT, Euroimmun, Lübeck, Germany), according to manufacturer instructions. In negative cases with a history strongly suggestive of NMOSD, an additional analysis was performed at the Neuroimmunology Laboratory of Innsbruck, using an in-house AQP4-transfected live cell assay [12]. The presence of MOG-Ab was analysed by three independent investigators (SF, SM, AF) at the Verona Neuropathology Laboratory using recombinant live cell-based immunofluorescence assay with HEK293A cells transfected with full-length MOG (human MOG alpha-1 EGFP fusion protein), as previously described [40].

Briefly, we performed the live cell staining immunofluorescence test 24 h after transfection. After a 10 min blocking step with goat IgG (Sigma-Aldrich) in PBS/10% FCS (both Sigma-Aldrich), cells were incubated with the serum samples diluted 1:20 and 1:40 in PBS/10% FCS for 1 h at 4 °C. After three washing steps with PBS, 10% FCS cells were incubated with CyTm 3-conjugated goat anti human IgG antibody (H+L, Jackson ImmunoResearch Laboratory, West Grove, PA, USA; diluted 1:200 in PBS/10% FCS) for 30 min at room temperature. Cells were washed twice and stained with DAPI (Sigma-Aldrich, diluted 1:10,000 in BS/10% FCS) to exclude dead cells, and immediately analysed with a fluorescence microscope (Zeiss, Axio Vert.A1). Only serial study numbers were provided and the clinical and radiological data were unknown to all the testing subjects. Sera were tested at dilutions of 1:20 and 1:40, and MOG-Ab positivity was titrated with serial dilutions with a threshold of 1:160 to define MOG-Ab positivity as previously established [40]. In patients positive for MOG-Ab who had available serum samples during the follow-up, repeated analysis of MOG-Ab was performed. To maximize analysis reliability, 157 samples (5 positive at antibody titre ≥ 1:160 and 152 negative) were analysed for MOG-Ab independently both at Verona Neuropathology and Innsbruck Neuroimmunology Laboratories. We established a high sensitivity assay for IgG subclass analysis to get an accurate picture of their distribution. The MOG-IgG subclass analysis of all samples was performed at the Neuroimmunology Laboratory of Innsbruck by CBA with specific secondary antibodies against the four IgG subclasses (Thermo Fisher, catalog number MH1015 for IgG1, 05-3500 for IgG2, 05-3600 for IgG3, MA5-16716 for IgG4) and revealed by fluorescence (Thermo Fisher, Alexa Fluor 594 goat anti-mouse IgG H+L). Screening at a dilution of 1:20 and 1:40 and titration of positive cases were performed. Results were interpreted by three independent investigators (MR, KS, SM) blinded to MOG-Abs results and clinical data. All MOG-Ab positive samples have been also tested using CD2-transfected cells and all of them were negative [40].

### Statistical analysis

Statistical analysis was performed using IBM SPSS, release V.24.0 (IBM Corporation). We compared clinical, demographic, MRI and serological data using the Mann–Whitney *U* test, Kruskal–Wallis test, Fisher’s exact test and Chi-square test. Statistical significance was defined as a two-sided *p* value of < 0.05. Inter-rater agreement was assessed by kappa statistics and correlations were analysed by Spearman’s correlations.

## Results

### Interlaboratory agreement

Agreement of the Verona and Innsbruck Laboratories for MOG-ab results (positive/negative) was 157/157 (100%), *κ* value = 1.000 (SE 0.000), *p* < 0.0001. Agreement of both laboratories for MOG antibody titres was 143/157 (91%), *κ* value = 0.692 (SE 0.067), *p* < 0.0001. All differences were only at one titre step, no differences affected positive/negative results. Titre levels of both laboratories showed a strong correlation (Spearman’s *ρ* = 0.911, SE 0.010, *p* < 0.0001).

### Demographic, clinical, and paraclinical data of MOG-Ab positive and negative cases

Demographic, clinical and radiological characteristics of the 154 analysed subjects are summarised in Table 1. Significant differences between the group of MOG-Ab positive and MOG-Ab negative patients were not observed in median age at onset, sex predominance, symptoms at onset and disease course. The median age at onset was 35 years in MOG-Ab positive cases, with a slight predominance of females (59%). MOG-Ab positive patients more often presented with isolated ON (59%) or isolated MY (36%), and less frequently with both ON and MY (4.5%). The onset was more heterogeneous in the MOG-Ab negative group, but ON (36%) and MY (54%) remained the most frequent manifestations at onset. Of note, 45% of MOG-Ab positive cases had infectious and flu-like prodromes, including fever, gastrointestinal symptoms, upper respiratory tract infection, and dental infection. In one case, a concomitant infection with both *Herpes virus 1* and *Borrelia Burgdorferi* was demonstrated, while in two patients a recent *Cytomegalovirus* infection was detected. Vaccination preceded the onset in one case. In both MOG-Ab seropositive and seronegative groups, the disease course was usually monophasic or relapsing, while a gradually evolving course was reported in one MOG-Ab positive patient and in nine MOG-Ab negative ones. ON was more frequently observed in MOG-Ab positive subjects compared with MOG-Ab negative cases, with a statistically significant difference, while the occurrence of myelitis was more frequent in MOG-Ab negative cases. No significant difference was observed in the rate of recovery. MOG-Ab positive cases usually reported only a partial recovery (77%), while only four patients experienced complete recovery. Statistically significant differences were noted among the final diagnoses in the two groups. The most frequently defined final diagnosis were MS (33%) and MY (32%) in seronegative subjects, while the seropositive ones mainly received a diagnosis of ON/CRION (45%) or other demyelinating disorders (23%) characterised by inflammatory conditions not included in other defined disorders. Interestingly, one MOG-Ab positive case received a definite diagnosis of MS according to the 2010 revision of McDonald criteria [34].

**Table 1:** Demographic, clinical and MRI data of MOG-ab positive and negative patients

	MOG-ab positive	MOG-ab negative	*p* value
Number of cases analysed	22	132	
Age at onset, median (range), years	35 (6–70)	36.5 (10–81)	0.540^a^
Female, % (*n*/total)	13 (59.1%)	79 (59.8%)	0.947^b^
Symptoms at onset			0.329^c^
ON	13 (59.1%)	47 (35.6%)	
Myelitis	8 (36.4%)	71 (53.8%)	
ON + myelitis	1 (4.5%)	1 (0.8%)	
Brainstem	0 (0%)	5 (3.8%)	
Cerebellum	0 (0%)	2 (1.5%)	
Brain	0 (0%)	3 (2.3%)	
ON + brain	0 (0%)	1 (0.8%)	
Other	0 (0%)	2 (1.5%)	
Disease course			0.671^c^
Monophasic	10 (45.5%)	70 (53%)	
Relapsing	11 (50%)	53 (40.2%)	
Gradually evolving	1 (4.5%)	9 (6.8%)	
Optic neuritis			0.001^c^
Never	6 (27.3%)	77 (58.3%)	
Unilateral	9 (40.9%)	37 (28%)	
Bilateral	5 (22.7%)	18 (13.6%)	
Unilateral and bilateral	2 (9.1%)	0 (0%)	
Myelitis	12 (54.5%)	94 (71.2%)	0.138^b^
Recovery			0.238^c^
Complete	4 (18.2%)	46 (34.8%)	
Partial	17 (77.3%)	77 (58.3%)	
No recovery	1 (4.5%)	9 (6.8%)	
Final diagnosis			<0.001^c^
CIS	0 (0%)	15 (11.4%)	
MS	1 (4.5%)	43 (32.6%)	
Myelitis	2 (9.1%)	42 (31.8%)	
ON	7 (31.8%)	22 (16.7%)	
CRION	3 (13.6%)	0 (0%)	
NMOSD	0 (0%)	4 (3%)	
ADEM	1 (4.5%)	0 (0%)	
ON + myelitis	3 (13.6%)	6 (4.5%)	
Other demyelinating disorders	5 (22.7%)	0 (0%)	
Follow-up, median (range), months	16.5 (1–276)	16.5 (1–288)	0.905^a^
Brain MRI			
Number of cases analysed	22	123	
Abnormal brain MRI	17 (77.3%)	97 (78.9%)	0.999^b^
Focal brain MS lesions			0.004^c^
None	14 (63.6%)	61 (50.0%)	
1–2	2 (9.1%)	2 (1.6%)	
3–4	5 (22.7%)	10 (8.1%)	
5–10	1 (4.5%)	32 (26%)	
> 10	0	18 (14.6%)	
Non-specific lesions	5 (22.7%)	32 (26.0%)	0.999^b^
Periventricular lesions	4 (18.2%)	58 (47.2%)	0.018^b^
Juxtacortical lesions	4 (18.2%)	51 (41.5%)	0.055^b^
Subtentorial lesions	7 (31.8%)	42 (34.1%)	0.999^b^
Optic nerve involvement			0.038^c^
None	15 (68.2%)	109 (88.6%)	
Unilateral	5 (22.7%)	11 (8.9%)	
Bilateral	2 (9.1%)	3 (2.4%)	
Spinal cord MRI			
Number of cases analysed	19	116	
Abnormal spinal cord MRI	11 (57.9%)	97 (83.6%)	0.025^b^
Short spinal cord lesions			0.362^c^
None	9 (47.4%)	36 (31.0%)	
One–two	4 (21.1%)	36 (31.0%)	
More than two	6 (31.6%)	44 (37.9%)	
LETM	2 (10.5%)	30 (25.9%)	0.242^b^
Cervical lesions	9 (47.4%)	77 (67.5%)	0.119^b^
Thoracic lesions	8 (42.1%)	54 (47.4%)	0.805^b^
Lumbar-conus lesions	4 (21.1%)	8 (7.0%)	0.070^b^
CSF data			
Number of cases analysed	18	112	
Cells number			0.072^b^
≤ 5 leukocytes/µL, % (*n*/total)	12 (66.7%)	95 (84.8%)	
5–20	2 (11.1%)	12 (10.7%)	
21–50	0	2 (1.8%)	
51–100	1 (5.6%)	0	
> 100	3 (16.7%)	3 (2.7%)	
Protein concentration > 45 mg/dL, % (*n*/total)	5 (27.8%)	11 (9.8%)	0.047^b^
IgG oligoclonal bands, % (*n*/total)			0.053^c^
Negative	12 (66.7%)	51 (45.9%)	
Positive	4 (22.2%)	56 (50.5%)	
Mirror pattern	2 (11.1%)	4 (3.6%)	

Groups were statistically compared with ^a^ Mann–Whitney *U* test, ^b^ Fisher’s exact test or ^c^ Chi-square test

*MOG*-*Ab* anti-myelin oligodendrocyte glycoprotein antibodies, *ON* optic neuritis, *CIS* clinically isolated syndrome, *MS* multiple sclerosis, *CRION* chronic relapsing inflammatory optic neuropathy, *NMOSD* neuromyelitis optica spectrum disorders, *ADEM* acute disseminated encephalomyelitis, *LETM* longitudinally extensive transverse myelitis, *CSF* cerebrospinal fluid

On brain MRI, 27% seropositive patients with available MRI data had more than two MS-like focal lesions, compared with 49% seronegative cases. However, 1–2 focal MS-lesions were more frequently observed in MOG-Ab positive cases (9 vs 2%). Periventricular and juxtacortical lesions were more frequently noted in seronegative patients (47 and 41.5%, respectively), while the subtentorial ones were equally distributed. ON involvement was predominant in MOG-Ab positive subjects (unilateral lesions in 23% of patients and bilateral in 9%). Statistically significant differences were noted in the number of MS-like lesions, periventricular distribution and ON involvement. Further details are reported in Table 1.

Spinal cord MRI resulted more frequently abnormal in MOG-Ab negative cases (84%) but no significant difference was noted in the two groups in length or location of lesions. In MOG-Ab positive subjects, spinal lesions were usually short (10 cases) with a predominant involvement of the cervical (47%) and thoracic (42%) regions.

Among CSF results, pleocytosis (> 5 leukocytes/µL) and increased protein concentration (> 45 mg/dL) were more frequently observed in seropositive cases (33 and 28%, respectively) compared to seronegative ones (15 and 10%, respectively), although only the latter reached statistical significance. Intrathecal IgG synthesis was predominant in MOG-Ab negative cases (50.5 vs 22%).

Further details of the 22 cases with positive MOG-Ab, including treatment and follow-up analysis are reported in the description of clinical cases in supplementary data.

### MOG-Ab and IgG subclass analysis

In seropositive cases, MOG-Ab was detectable at different titres with a range of 1:160–1:81,920. Longitudinal serum samples were available in 6 of 22 MOG-Ab positive cases and were analysed for MOG-IgG subclasses (Table 1 supplementary data). MOG-Ab titres usually decreased in non-relapsing cases, regardless the partial or complete recovery, and could fall below the cut-off even few months after the acute stage.

All MOG-IgG positive patients (≥ 1:160) were reactive for IgG1 antibodies, which were the predominant subclass in 19/22 cases. Out of these, 12/22 subjects were exclusively positive for IgG1 subtype (2 ON + MY, 6 ON, 4 other demyelinating disorders). An equal amount of IgG subclasses was detected in three patients with reactivity to IgG1/IgG3/IgG4 (MS), IgG1/IgG2/IgG3 (ON) and all IgG subclasses (ON). One patient presented with all the subclasses, however, IgG2/IgG3/IgG4 had a lower titre than IgG1 (other demyelinating disorders). IgG3 and IgG2 were also observed in four cases (two with MY, one with ON, one with ADEM) and one case (ON), respectively, but also in these cases, IgG1 had higher titre. One case presented both IgG2 and IgG3 at lower titre than IgG1. We also detected IgG subclass antibodies in 12 MOG-Ab seronegative cases: IgG1 (8/12), IgG2 (1/12) and IgG3 (3/12), due to high sensitivity of the assay (Tables 2, 3). Among these patients, five subjects received a final diagnosis of CIS, four of MY, one of ON, one of NMOSD and one of MS. Details of cases tested negative for MOG-IgG but positive for the IgG subclasses are reported in Table 3.

**Table 2:** MOG-IgG positive cases in the analysed cohort, according to total IgG and IgG subclasses

	MOG-IgG (H + L) positive	MOG-IgG (H + L) negative
Number of cases	22	132
MOG-IgG (H + L) titre (median, range)	640 (160–81,920)	0 (0–80)
MOG-IgG1 seropositive	22 (100%)	8 (6.1%)
MOG-IgG1 titre (median, range)	640 (20–10,240)	0 (0–80)
MOG-IgG2 seropositive	5 (22.7%)	1 (0.8%)
MOG-IgG2 titre (median, range)	0 (0–320)	0 (0–20)
MOG-IgG3 seropositive	9 (40.9%)	3 (2.3%)
MOG-IgG3 titre (median, range)	0 (0–640)	0 (0–40)
MOG-IgG4 seropositive	3 (13.6%)	0 (0%)
MOG-IgG4 titre (median, range)	0 (0–640)	0 (0)

**Table 3:** Analysis of cases tested seronegative for MOG-IgG but positive for MOG-IgG1, IgG2 or IgG3

Case	Sex	Age	Onset	Final diagnosis	MOG antibody titer (1:)				
					IgG (H + L)	IgG1	IgG2	IgG3	IgG4
1	M	20	ON	MS	0	40	0	0	0
2	M	31	MY	CIS	0	40	0	0	0
3	F	33	ON	CIS	0	20	0	0	0
4	F	35	ON + MY	CIS	40	80	0	0	0
5	F	65	Brainstem	NMOSD	0	20	0	0	0
6	F	30	MY	MY	40	40	0	0	0
7	M	36	ON	ON	80	20	0	0	0
8	M	49	MY	CIS	0	20	0	0	0
9	F	55	MY	MY	0	0	0	20	0
10	F	31	MY	MY	80	0	0	40	0
11	F	28	MY	CIS	40	0	0	20	0
12	M	63	MY	MY	80	0	20	0	0

*MOG*-*Ab* anti-myelin oligodendrocyte glycoprotein antibodies, *M* male, *F* female, *ON* optic neuritis, *MY* myelitis, *MS* multiple sclerosis, *CIS* clinically isolated syndrome, *NMOSD* neuromyelitis optica spectrum disorders

## Discussion

**Fig. 2:**
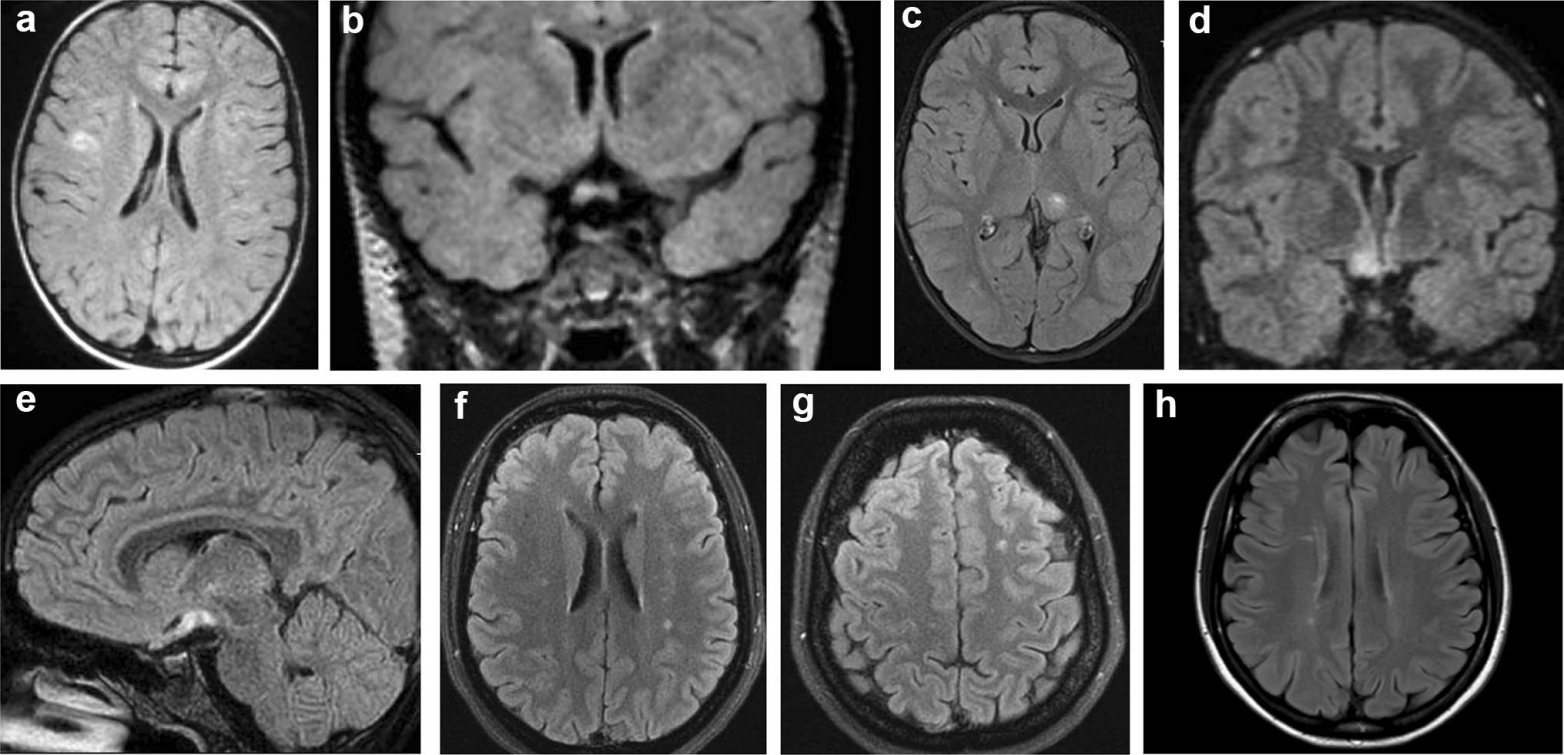
Brain radiological findings. One isolated frontal lesion (**a**), followed by monolateral optic nerve (**b**) and thalamic involvement (**c**) and subsequently by chiasmatic and monolateral optic tract damage (**d**, **e**) are shown in case 18. Non-specific white matter lesions were observed at onset in case 13 (**f**, **g**) while typical white matter lesions are shown in the patient with MS diagnosis (case 22, **h**)

We here report serological, MRI, and CSF features of 22 patients affected with IDD who resulted seropositive for MOG-Ab and compare them with a group of seronegative cases, with the aim of investigating the diagnostic and prognostic utility of this immunological marker. Comparing MOG-Ab positive and negative cases, we observed that ON, either unilateral, bilateral or both, as well as radiological involvement of the optic nerve, were predominant in MOG-Ab positive cases, suggesting that ON is a crucial feature of this condition. Another brain MRI hallmark was the predominant presence of 1–2 MS-like lesions in seropositive patients. However, brain MRI findings were very heterogeneous (Fig. 2), and usually not diriment for the final diagnosis in MOG-Ab positive cases. Moreover, MOG-Ab positive subjects frequently showed normal spinal cord MRI or only short MS-like spinal cord lesions. Taken together, these aspects underline the relevance of ON involvement in MOG-positive cases but also the difficult clinical and radiological characterisation of this condition. This could explain why some MOG-Ab positive patients received a final diagnosis of “other demyelinating disorders” defined by the evidence of an inflammatory disorder not fulfilling diagnostic criteria for MS, NMOSD, or ADEM. In the seropositive group, we observed a slight female preponderance, which is partially discordant from previously reported data [24, 30, 41], while the median age at onset was in line with previous findings [26, 30]. We also noted a high percentage of feverish prodromes during the first attack, although a concomitant infection was demonstrated only in few cases. These findings suggest that a definite or unknown pathogen could act as a self-mimic agent leading to direct damage and further activation of the immune system via epitope spreading or bystander activation caused by the inflammatory environment [42].

In the present study, MOG-Ab positive cases had overall clinical features in line with previous reports, indicating the optic nerve and the spinal cord as the preferential anatomical sites clinically involved at the onset and during the follow-up [21, 26, 30, 43–49]. However, we did not observe a phenotype compatible with the criteria of NMOSD among our cohort of MOG-Ab positive cases, which is partially discordant with data recently reported [26, 30, 49, 50].

Of note, we also observed MOG-Ab in a patient with definite diagnosis of MS characterised by ON at onset, recurrent myelitis, CSF restricted OB and typical brain and spinal cord MRI features. These data are in accordance with previously reported findings on MOG-Ab positivity in preselected patients with MS and in subjects with biopsy-proven MS type II pathology [18, 27, 50]. However, in our case we did not observe the severe brainstem and spinal cord involvement previously reported and the disease course was more benign. Interestingly, this subject resulted positive for total MOG-IgG (titre 1:160), IgG1 (titre 1:20), IgG3 (titre 1:40), and also IgG4 subclass (titre 1:20). All together these findings confirm that MOG-Ab can be detected also in patients with typical MS features and that the spectrum of MOG-associated disorders is probably wider than expected.

Compared to some of the results previously reported [26, 30, 49, 50], we noted a higher proportion of MOG-Ab positive patients with a monophasic course, even if relapses were frequently observed. Of note, the observation time was shorter in subjects with a single attack (median 9 months) compared to those with a relapsing course (median 30 months). Even if relapses seem to occur within the first months [49], we cannot rule out a relapsing course in some of the monophasic MOG-Ab positive patients upon further follow-up. Conversely, the proportion of cases with neurological *sequelae* after the acute attack was higher in our patients compared to other studies [16, 17, 30, 49], suggesting that MOG-Ab do not denote a benign entity.

Several previous studies reported increased protein concentration and pleocytosis in the absence of OB on CSF analysis in most of these patients [26, 51–53]. We also noted pleocytosis, increased proteins but also intrathecal IgG synthesis in MOG-Ab positive cases, suggesting that CSF restricted OB cannot define the differential diagnosis of seropositive and seronegative cases.

In our study, MRI findings are similar to those observed in previous studies of MOG-Ab positive cases showing normal brain MRI, unspecific findings, prominent optic nerve involvement, but also other abnormalities resembling those observed in subjects with MS or NMOSD with AQP4-Ab [21, 25, 45, 54]. According to previously reported data, we also noted the prevalence of few brain lesions (< 2) in patients with MOG-Ab compared to seronegative ones [55]. However, the spinal cord MRI data in our group of patients did not confirm the high prevalence of LETM lesions previously reported in patients with serum MOG-Ab [26, 41, 49, 51, 54]. Although other authors described cases with spinal cord lesions extending less than three vertebral segments on MRI [30], we observed a higher proportion of short lesion, especially at the onset. Even if a referral bias cannot be excluded, this observation underlines the possible role of MOG-Ab in patients with short myelitis, which could be a crucial point in the clinical setting.

Another interesting observation is that MOG-Ab titre decrease and also fall below cut-off in non-relapsing patients, despite partial or complete recovery, while tend to increase during relapses. Even if results about longitudinal analysis of MOG-Ab are contradictory [20, 27, 43, 47, 56–58], our observations are in line with recently reported data on the association between negative serological conversion and a benign clinical course [11, 50]. These findings underline the importance of testing patients during the acute phase due to the possibility of negative serological conversion even in a few months, and encourage to follow-up MOG-Ab titre during the course of the disease.

The observation of the mostly predominant subclass of IgG1 in MOG-Ab positive cases suggests that both anti-total IgG and -IgG1 based assays could give comparable results in detecting positive cases. We were also able to detect the IgG subclass presence is some of the total IgG negative cases, and this is due to the high sensitivity for which our IgG subclass test was designed. These findings are in accordance with previously reported data indicating that the IgG1 assay could identify also patients below the cut-off for total IgG [19]. Actually, three cases with a suggestive phenotype, with a final diagnosis of NMOSD, ON and MY, respectively, were identified by IgG1 only. However, also four cases with a final diagnosis of CIS and one with a diagnosis of MS had isolated IgG1, suggesting a possible loss of specificity. The observation that isolated IgG3 and IgG2 were detectable in two cases and one case, respectively, supports the high sensitivity of this test. Since these subtypes and, in particular, IgG3 are able to initiate cell-mediated cytotoxicity and to fix complement, their role in inducing inflammatory conditions cannot be excluded.

Our study is limited by several factors. We analysed patients who have been referred to our laboratory for antibodies testing and this resulted in an unintentional inclusion bias that cannot be completely avoided and could influence the profile of included patients. This selection bias is partially reduced by the multicentre design of the study, including 16 academic centres, and by the recruitment among both inpatients and outpatients. Then the study was in part retrospective and this did not allow standardizing the collection of variables and timing of procedures. In addition, it was not possible to monitor MOG-Ab titre and MRI in all subjects over time on a regular basis. Finally, the follow-up duration was relatively short, possibly limiting the validity of classifying patients accordingly to the relapsing or monophasic course. Beside these limitations, the report of MOG-Ab positive cases and the comparison with seronegative ones provide interesting data on the meaning of MOG-Ab in the clinical setting. Moreover, the extensive analysis of IgG subclasses and the comparison of different tests are relevant for the interpretation of MOG-associated pathology.

## Electronic supplementary material

Below is the link to the electronic supplementary material.

Supplementary material 1 (DOCX 228 kb)

Supplementary material 2 (PDF 111 kb)
