# Supplementary material for: Clinical spectrum and IgG subclass analysis of anti-myelin oligodendrocyte glycoprotein antibody-associated syndromes: a multicenter study
Source: J Neurol. 2017 Oct 23;264(12):2420–30. doi: 10.1007/s00415-017-8635-4 (PMC5688213; doi:10.1007/s00415-017-8635-4)
Supplement: Supplementary file 1 — Supplementary material 1 (DOCX 228 kb) [file 415_2017_8635_MOESM1_ESM.docx]

**Clinical spectrum and IgG subclass analysis of anti-myelin oligodendrocyte glycoprotein antibody-associated syndromes: a multicenter study**

*Journal of Neurology*

Sara Mariotto, Sergio Ferrari, Salvatore Monaco, Maria Donata Benedetti, Kathrin Schanda, Daniela Alberti, Alessia Farinazzo, Ruggero Capra, Chiara Mancinelli, Nicola De Rossi, Roberto Bombardi, Luigi Zuliani, Marco Zoccarato, Raffaella Tanel, Adriana Bonora, Marco Turatti, Massimiliano Calabrese, Alberto Polo, Antonino Pavone, Luisa Grazian, GianPietro Sechi, Elia Sechi, Daniele Urso, Rachele Delogu, Janes Francesco, Luciano Deotto, Morena Cadaldini, Maria Rachele Bianchi, Gaetano Cantalupo, Markus Reindl, Alberto Gajofatto

Corresponding author:

Sara Mariotto

[sara.mariotto@gmail.com](mailto:sara.mariotto@gmail.com)

Department of Neuroscience, Biomedicine and Movement Sciences,

University of Verona, Verona, Italy

Policlinco GB Rossi, P.le LA Scuro 10, 37134, Verona, Italy

Tel. +39 0458124461

**Table 1. Supplementary material**

MOG-Ab titre and subclass analysis in patients with follow-up samples

* During remission

# During relapse
