# Supplementary material for: Clinical spectrum and IgG subclass analysis of anti-myelin oligodendrocyte glycoprotein antibody-associated syndromes: a multicenter study
Source: J Neurol. 2017 Oct 23;264(12):2420–30. doi: 10.1007/s00415-017-8635-4 (PMC5688213; doi:10.1007/s00415-017-8635-4)
Supplement: Supplementary file 2 — Supplementary material 2 (PDF 111 kb) [file 415_2017_8635_MOESM2_ESM.pdf]

Clinical spectrum and IgG subclass analysis of anti-myelin oligodendrocyte glycoprotein antibody-associated syndromes: a multicenter study

Journal of Neurology

Sara Mariotto, Sergio Ferrari, Salvatore Monaco, Maria Donata Benedetti, Kathrin Schanda, Daniela Alberti, Alessia Farinazzo, Ruggero Capra, Chiara Mancinelli, Nicola De Rossi, Roberto Bombardi, Luigi Zuliani, Marco Zoccarato, Raffaella Tanel, Adriana Bonora, Marco Turatti, Massimiliano Calabrese, Alberto Polo, Antonino Pavone, Luisa Grazian, GianPietro Sechi, Elia Sechi, Daniele Urso, Rachele Delogu, Janes Francesco, Luciano Deotto, Morena Cadaldini, Maria Rachele Bianchi, Gaetano Cantalupo, Markus Reindl, Alberto Gajofatto

Corresponding author:

Sara Mariotto

sara.mariotto@gmail.com

Department of Neuroscience, Biomedicine and Movement Sciences,

University of Verona, Verona, Italy

Policlinco GB Rossi, P.le LA Scuro 10, 37134, Verona, Italy

Tel. +39 0458124461

Report of clinical, radiological, serological, CSF data and follow-up analysis of the 22 MOG-Ab positive cases

Case 1.

A 49-year-old woman presented with bilateral visual impairment. On brain MRI a bilateral involvement of the optic nerve was noted, while spinal cord MRI was normal. Standard CSF examination was negative, and OB were absent. An extensive autoimmunity blood work-up was negative, while MOG-Ab tested in the acute stage resulted positive at 1:320 dilution. Visual evoked potential (VEP) were delayed in latency and reduced in amplitude bilaterally. Partial improvement was reported after high dose intravenous (i.v.) steroids. At last evaluation 12 months after onset the clinical status was unchanged (visual acuity 0.6 in the right eye and 0.8 in the left; EDSS: 2.0).

Case 2.

A 70-year-old man presented with subacute lower limbs weakness. Brain MRI was normal while spinal cord MRI showed a thoracic lesion shorter than three vertebral segments, suggestive of an inflammatory area (lesion appearance was not consistent with spinal cord infarction). CSF analysis resulted negative. Subsequent follow-up up to 5 years after onset showed no clinical improvement. MOG-Ab tested 5 years after the onset were positive at 1:160. The EDSS at the last follow-up was 4.0.

Case 3.

A 25-year-old woman reported a subacute scotoma in the right eye. VEP were delayed in latency on the right side while brain MRI was normal. On spinal cord MRI a cervical lesion at C2 level without contrast enhancement was noted. The patient refused lumbar puncture. MOG-Ab tested in the acute stage resulted positive at a titre of 1:320. After high dose steroids the clinical picture remained unchanged. The patient received additional treatment with an i.v. immunoglobulins course, followed by high-dose i.v. cyclophosphamide. Subsequently, glatiramer acetate was started. At the last follow-up, 11 months after the onset, the patient reported a psychotic episode. Brain MRI showed three T2-hyperintense infratentorial, juxtacortical and periventricular lesions not suggestive of MS. EDSS observed at the last follow-up was 2.0.

Case 4.

A 39-year-old woman reported an episode of right optic neuritis associated with Lhermitte’s sign, which completely resolved after steroid therapy. Brain MRI showed punctuate T2-hyperintensities in the periventricular white matter and in the optic nerve bilaterally, not suggestive of MS lesions. Spinal cord MRI was negative. CSF examination and blood work-up were negative, except for reduction of thyroid-stimulating hormone (TSH)

plasma level. VEP were delayed in P100 latency bilaterally. One year later, a spinal cord MRI showed a right paramedian T2-hyperintense lesion at C5-C6 level with a longitudinal extension of 8 mm. Two additional non-enhancing lesions were observed in the right pons and left frontal region on a brain MRI performed three years after the initial event. Subsequently, the patient had a left optic neuritis. MOG-Ab tested during the relapse resulted positive at 1:160 dilution and the patient was started on azathioprine, which was interrupted few weeks later due to side effects. MOG-Ab titre resulted negative at a control performed 8 and 13 months later (titre 1:20 and 0 respectively). Only a partial improvement was noted at the last follow-up (EDSS: 1.0).

Case 5.

A 51-year-old woman had a history of bilateral visual loss at age 29, which responded to steroid treatment. After that, she experienced relapsing episodes of monolateral and bilateral visual impairment, gait imbalance, urinary retention, and numbness in the lower limbs over several years, which partially recovered with steroids. Brain and spinal cord MRI, CSF examination, and extensive blood work-up for differential diagnosis were repeatedly normal. VEP showed delayed P100 latency bilaterally. When the patient was 49-year-old she developed subacute paraparesis, which did not recovered. Two years later, speech and behavioral changes were noted. At that time MOG-Ab resulted positive with a titre of 1:1280. CSF and spinal cord MRI were again normal, while brain MRI showed bilateral periventricular and juxtacortical confluent lesions with no evidence of contrast enhancement. Diffuse atrophy was also noted. The EDSS at the last follow-up was 5.5.

Case 6.

A 31-year-old woman experienced fever, gastrointestinal symptoms and headache followed by lower limb weakness, numbness and bladder dysfunction. Serological analysis revealed a recent infection with *Herpes virus* and *Borrelia burgdorferi* and antinuclear antibodies (ANA) positivity (1:320). Pleocytosis (150 cells/µL, 70% mononuclear), increased proteins (74 mg/dl), and mirror OB pattern were observed on CSF analysis. Brain MRI resulted negative while spinal cord imaging showed multiple thoracic non-extensive and conus lesions. A repeated CSF analysis showed normal protein levels and persistence pleocytosis (30 cells/µL). On MRI lesions in medulla oblongata and cerebellar peduncles were noted in association with cervical extensive lesion and new dorsal non-extensive lesions. Suspecting an infectious aetiology, the patient was initially treated with antibiotics and acyclovir but worsening of the clinical picture was observed. MOG-Ab resulted positive at a titre of 1:640. Treatment with steroids and plasma exchange were started. A reduction of both brain and spinal cord lesions and a partial clinical improvement was noted at the last evaluation, 2 months later (EDSS: 3.5).

Case 7.

A 57-year-old man developed headache and unilateral visual loss. Non-specific subcortical and white matter lesions were present on brain MRI while spinal cord MRI was negative. After treatment with steroids a complete recovery was obtained. Eight months later, monolateral visual loss appeared with partial improvement after steroids. Eighteen months later vision deteriorated again. Brain and spinal cord MRI were unchanged.
VEP were not recordable unilaterally and CSF analysis resulted negative. The patient was treated with antiplatelet drugs for a presumptive diagnosis of ischemic optic neuritis. Three years later bilateral optic neuritis occurred and a diagnosis of CRION was made. Brain MRI resulted unchanged and bilateral delay in P100 latency with monolateral reduction of amplitude was observed. One month later monolateral optic neuritis occurred with partial improvement after steroids therapy. The patient was started on azathioprine and then methotrexate, both interrupted due to side effects. Chronic steroids (5 mg/die) were then administered for two years. MOG-Ab resulted positive at a titre of 1:2560. At last follow-up clinical examination demonstrated visual acuity (VA) of 4/10 in the right eye and 9/10 in the left eye (EDSS: 2.0).

Case 8.

A 34-year-old woman experienced lower limbs pain, perineal sensory impairment, and bladder dysfunctions one month after gastrointestinal symptoms. A brain MRI resulted negative while spinal cord imaging revealed cervical, thoracic, and lumbar non-extensive lesions. CSF examination disclosed pleocytosis (60 cells/µL) and increased proteins (49 mg/dl) in absence of OB; VEP resulted negative. MOG-Ab were present at a titre of 1:160. A complete recovery was observed after steroids administration with a complete disappearance of spinal cord lesions. Four, 6, and 9 months after the onset MOG-Ab titre decreased to 0, 1:40, and 0 respectively.

Case 9.

A man developed gastrointestinal symptoms followed by bladder dysfunction, lower limb weakness and numbness at the age of 40. While brain MRI revealed a small enhanced lesion in the pons, spinal cord imaging showed cervical, thoracic, lumbar and conus non-extensive lesions. CSF analysis showed pleocytosis (180 cells/µL, prevalence of mononuclear) and increased proteins (124 mg/dl) in absence of CSF-restricted OB. MOG-Ab were present at a titre of 1:81.920. The patient was given antibiotics associated with acyclovir and steroids and then, for the progression of symptoms, plasma exchange. Treatment resulted in almost complete recovery and a radiological study performed 8 months after the onset showed the remission of the pre-existing lesions. Only slight bladder dysfunctions and lower limbs sensory impairment were documented at the last evaluation, 4 years later (EDSS: 2.0). MOG-Ab retested at this moment resulted negative (titre 1:80).

Case 10.

A young woman experienced bilateral visual loss and ocular pain associated with headache at the age of 15, three weeks after a gastrointestinal infection. On serological analysis a recent *Cytomegalovirus* infection emerged. CSF examination was negative while brain MRI noted sinusitis and bilateral involvement of the ON, in particular in the pre-orbital and retro-orbital region with contrast enhancement. On neurological evaluation papillitis with swelling of the optic disc, blurring of disc margins and one perivascular hemorrhage emerged. MOG-Ab resulted positive at a titre of 1:640. The patient was treated with i.v. steroids with a prompt recovery. Oral steroids were given for the next month. At the last evaluation visual acuity was 20/20 in the right eye and 20/25 in the left eye (EDSS: 1.0). A control brain MRI confirmed a bilateral involvement of the optic nerve with signs of atrophy, while spinal cord MRI was negative.

Case 11.

A 36-year-old man developed fever, lower limbs sensory impairment, bowel, bladder and sexual dysfunctions. A brain MRI noted lesion in the pons, cerebellar peduncle, and periventricular regions while spinal cord MRI showed cervical, thoracic, lumbar and conus non-extensive lesions. A recent *Cytomegalovirus* infection emerged from serum analysis and CSF documented pleocytosis (16 cells/µL), increased proteins (80 mg/dl) and CSF-restricted OB. Suspecting an infection disorder the patient was given antibiotics and acyclovir and almost complete improvement was noted. Five year later the patient reported a progression of the clinical symptoms (EDSS: 2.5) so that a complete autoimmune panel was tested. MOG-Ab resulted positive at a titre of 1:160 and treatment with azathioprine was proposed.

Case 12.

A woman with a history of transient episodes of upper limbs numbness and bladder dysfunction presented unilateral visual loss at the age of 43. VEP confirmed ON and the involvement of the optic nerve was also visible on brain MRI. CSF analysis resulted negative. During ON, MOG-Ab were present at a titre of 1:160 and the patient was given steroids. Symptoms partially remitted at the last evaluation, 3 months later (EDSS: 2.0).

Case 13.

A 50-year-old woman experienced unilateral visual loss. A first brain MRI noted only non-specific white matter and periventricular lesions while VEP resulted delayed in latency unilaterally. CSF analysis showed a mirror OB pattern. Treatment with steroids resulted in almost complete recovery (VA of 9/10 in the right eye and 10/10 in

the left eye). Three attacks of ON occurred in the subsequent 8 months, the last episode associated with ocular pain. In this occasion brain MRI noted retrobulbar optic nerve involvement and MOG-Ab resulted positive at a titre of 1:640. Treatment with steroids resulted only in partial improvement (EDSS: 2.0).

Case 14.

A man developed fever and sinusitis followed by unilateral visual loss and ocular pain at the age of 30. Brain and spinal cord MRI resulted negative. MOG-Ab resulted positive at a titre of 1:2560 and a diagnosis of papillitis was made by the ophthalmologist. Treatment with steroids and antibiotics resulted in only partial recovery 2 months later (EDSS: 2.0).

Case 15.

A 23-year-old man presented with transient unilateral visual loss and ocular pain. The day after a second attack of unilateral persistent visual loss (VA 3/10) and ocular pain occurred. Brain MRI demonstrated only sinusitis and a small frontal cavernous angioma, while spinal cord imaging was negative. Involvement of nasal fibers of the optic nerve was observed on VEP (N105 component). Routine and autoimmune screening resulted negative except for the detection of MOG-Ab at a titre of 1:320. The patient was given high dose i.v. steroids followed by oral tapering and symptoms partially improved (VA 5/10 in the affected eye; EDSS: 2.0).

Case 16.

A 55-year-old man experienced bilateral visual loss and ocular pain. On neurological examination only visual impairment (VA 0/10 in the right eye and 2/10 in the left eye) was noted. Brain MRI demonstrated one non-specific white matter lesion, while VEP resulted not recordable bilaterally. Among the serological evaluation, MOG-Ab resulted positive at a titre of 1:10240. The patient was treated with steroids that resulted in almost complete improvement. Three months later MOG-Ab titre resulted of 1:320 and the clinical picture was unchanged (EDSS: 1.0).

Case 17.

A man presented with fever, upper respiratory tract infection and gastrointestinal symptoms followed by upper and lower limbs dysesthesia, lower limbs pain, and bladder dysfunction at the age of 23. Spinal cord MRI demonstrated an extensive cervical lesion associated with non-extensive thoracic ones. Brain MRI showed only non-specific subcortical lesions. Pleocytosis (180 cells/μL, mainly mononuclear), increased proteins (49 mg/dl),

and the presence of CSF-restricted OB were noted on CSF evaluation. MOG-Ab resulted positive at a titre of 1:1280. The patient received steroids with slow tapering and then azathioprine that resulted in complete remission. A reduction of the pre-existing lesions, disappeared after few months, was observed on spinal cord MRI. The patient reported symptom fluctuation in the subsequent 2 years, sometimes after upper respiratory tract infection. A titre of 1:320 of MOG-Ab was detected 30 month after the onset, during complete recovery.

Case 18.

A child developed gastrointestinal symptoms followed by bilateral visual loss and dysarthria at the age of 6. Only one lesion in the frontal region was observed on brain MRI. Symptoms spontaneously disappeared. One month later monolateral ocular pain and bilateral visual loss appeared (VA 1/10 in the right eye and 2/10 in the left eye). One new parietal lesion associated with monolateral optic nerve and chiasmatic involvement with contrast enhancement was observed on brain MRI. CSF analysis resulted negative while VEP were delayed in latency bilaterally. The patient received steroids and experienced a complete recovery (VA 10/10). New subcortical and one thalamic lesions were observed few month later on brain MRI, which subsequently disappeared at a new control. Five years later the patient experienced bilateral ON associated with ocular pain (VA 5/10 in the right eye and 6/10 in the left eye). Chiasmatic and monolateral optic tract involvement in absence of the pre-existing lesions was noted on brain MRI while spinal cord study resulted negative. Steroids with slow tapering were administered, leading to a complete recovery in one month. A new attack of ON took place 6 months later. During this last episode, MOG-Ab resulted positive at a titre of 1:320. We tested also the sample obtained at onset, which resulted positive, at the same titre. Treatment with steroids and plasma exchange led to partial recovery (VA 6/10) and a decrease of MOG-Ab titre (1:40). Mycophenolate mofetil was then started. The EDSS at the last follow-up was 2.0.

Case 19.

A 37-year-old man presented with fever after influenza vaccination followed by lower limb numbness and weakness, abdominal dysesthesia, sexual and bladder dysfunctions, and Lhermitte’s sign. Brain and spinal cord MRI and CSF analysis were negative. Treatment with steroids resulted in a gradual complete recovery. An attack of fever followed by lower limbs numbness, sexual dysfunctions and monolateral visual impairment (VA 5/10) occurred 13 years later. One corpus callosum and one cerebellar peduncle lesion associated with non-extensive dorsal lesions were detected on radiological evaluation. VEP resulted delayed in latency bilaterally. MOG-Ab

resulted positive at a titre of 1:640. Treatment with steroids led to a complete recovery and azathioprine was then started.

Case 20.

A woman experienced monolateral visual loss at the age of 57. Treatment with steroids led to a complete regression of the symptoms in two weeks. Few weeks later a new attack of ON occurred, confirmed by bilateral delayed of P100 latency on VEP. CSF restricted OB were detected on CSF analysis. Brain MRI was negative while signs of pre-existent myelitis in the cervical end thoracic regions were noted on spinal cord study. MOG-Ab were present at a titre of 1:640. Treatment with steroids resulted in a partial recovery (EDSS: 1.0).

Case 21.

A 34-year-old woman presented with monolateral visual loss and bilateral ocular pain. Treatment with steroid resulted in complete recovery in one week. One month later a new attack of ON occurred and symptoms completely remitted after 1 week of steroids therapy. Ten days after steroids discontinuation ON associated with ocular pain occurred. CSF analysis was negative as brain and spinal cord MRI, while VEP showed increased P100 latency unilaterally. Symptoms almost completely remitted after steroids therapy. A new attack of ON occurred 5 days after steroids discontinuation. Almost complete recovery was achieved after steroids administration and mycophenolate mofetil was added because of muscular pain associated with ANA positivity (1:160). Two years after the onset ocular pain occurred and brain MRI confirmed distal ON involvement. MOG-Ab resulted positive at a titre of 1:640. The patient was treated with steroids that resulted in only partial improvement (EDSS: 2.0).

Case 22.

A 19-year-old woman developed unilateral visual loss (VA 6/10) followed by lower limbs sensory impairment and Lhermitte’s sign. A brain MRI noted only one small lesion near the corpus callosum. A complete spontaneous recovery was noted after one month. After 15 years, a progressive sensory and motor involvement of the right arm followed by monolateral lower limb sensory impairment was reported. A brain MRI demonstrated multiple non-enhancing lesions involving corpus callosum and the periventricular region, pons, and cerebellar peduncle. A spinal cord MRI noted multiple non-extensive cervical lesions in absence of contrast enhancement. On CSF analysis a slight increase of cells (10 cells/μ) and the presence of CSF restricted OB were observed. MOG-Ab resulted positive at a titre of 1:160. The patient was treated with i.v. steroids followed by oral tapering and symptoms partially improved. A diagnosis of MS was made and treatment with interferon beta-

1a was started. Due to side effects the patient was then given glatiramer acetate. A new attack of monolateral upper limb sensory and motor impairment occurred 8 months later but a control spinal cord MRI was unchanged. One month later the clinical picture was partially improved with persistence of slight distal upper limb sensory and motor impairment (EDSS: 2.5).
